# The St. Louis Polyclinic

**Published:** 1889-06

**Authors:** 


### New Journals.

The St. Louis Polyclinic is the name of the official organ of the St. Louis Post-Graduate School of Medicine, which is edited in conjunction with the Faculty by L. A. Turnbull, M. D. The first number appeared in April of this year, and is a creditable one. We extend it a cordial greeting.

